# MuPeG—The Multiple Person Gait Framework

**DOI:** 10.3390/s20051358

**Published:** 2020-03-02

**Authors:** Rubén Delgado-Escaño, Francisco M. Castro, Julián R. Cózar, Manuel J. Marín-Jiménez, Nicolás Guil

**Affiliations:** 1 Department of Computer Architecture, University of Málaga, 29071 Málaga, Spain; fcastro@uma.es (F.M.C.); julian@uma.es (J.R.C.); nguil@uma.es (N.G.); 2 Department of Computer Science and Numerical Analysis, University of Córdoba, 14071 Córdoba, Spain; mjmarin@uco.es

**Keywords:** gait recognition, gait framework, gait dataset, multiple subjects, augmented dataset

## Abstract

Gait recognition is being employed as an effective approach to identify people without requiring subject collaboration. Nowadays, developed techniques for this task are obtaining high performance on current datasets (usually more than 90% of accuracy). However, those datasets are simple as they only contain one subject in the scene at the same time. This fact limits the extrapolation of the results to real world conditions where, usually, multiple subjects are simultaneously present at the scene, generating different types of occlusions and requiring better tracking methods and models trained to deal with those situations. Thus, with the aim of evaluating more realistic and challenging situations appearing in scenarios with multiple subjects, we release a new framework (MuPeG) that generates augmented datasets with multiple subjects using existing datasets as input. By this way, it is not necessary to record and label new videos, since it is automatically done by our framework. In addition, based on the use of datasets generated by our framework, we propose an experimental methodology that describes how to use datasets with multiple subjects and the recommended experiments that are necessary to perform. Moreover, we release the first experimental results using datasets with multiple subjects. In our case, we use an augmented version of TUM-GAID and CASIA-B datasets obtained with our framework. In these augmented datasets the obtained accuracies are 54.8% and 42.3% whereas in the original datasets (single subject), the same model achieved 99.7% and 98.0% for TUM-GAID and CASIA-B, respectively. The performance drop shows clearly that the difficulty of datasets with multiple subjects in the scene is much higher than the ones reported in the literature for a single subject. Thus, our proposed framework is able to generate useful datasets with multiple subjects which are more similar to real life situations.

## 1. Introduction

Nowadays, the presence of video-surveillance cameras in our cities is common and they are considered as one more element of the street furniture. Images and videos recorded with those cameras can be used for people identification in video-surveillance applications. Three of the main challenges of those images and videos are the distance from which they are recorded, the occlusions produced by people and street furniture, and the presence of multiple people in the scene. Therefore, effective people identification approaches must deal with these problems.

Typically, the problem with the distance has been faced using *gait recognition*, which is a kind of biometric pattern that identifies people by the way they walk. In contrast to commonly used biometric patterns such as iris- or fingerprint-based approaches [1,2], gait-based methods can be considered as non-invasive since they can be performed at a distance and do not require the cooperation of the subjects that have to be identified. Thus, gait recognition can be used in the context of video surveillance to control the access of people to special areas or identify dangerous people at certain distance. Many approaches have been proposed for solving the problem of identifying people based on gait recognition [3]. However, most of them do not solve the last two challenges commented above so that their applicability to real-life scenarios is limited.

Regarding occlusions, some works [4,5,6] have developed mechanisms to alleviate this problem in the context of gait recognition. In most of the cases, those approaches only solve partial issues like improving incorrect subject segmentation or reducing the noise produced during recording time. Although those approaches try to solve occlusions produced by static objects, the occlusions produced by dynamic objects, like other people, is still an unsolved issue. In addition, this problem has not been treated in detail because most of gait datasets publicly available have been recorded in controlled situations without occlusions or with very limited kind of occlusions that are far from real situations.

Concerning gait recognition when several people are simultaneously walking in the scene, there are no works trying to identify multiple subjects at the same time. Like in the previous challenge, it is due to the absence of realistic datasets with multiple subjects walking freely in the scene. Currently, there exists a dataset [7] with multiple subjects in the scene but, the recognition process is focused on the main subject, which is occluded by other static people. Thus, they only focus on occlusions but not on the identifying of all subjects in the scene, which is a much more challenging problem that requires accurate people detection and tracking methods to produce good results.

Therefore, there is a significant lack of realistic datasets in the gait recognition field. In order to solve this absence of datasets dealing with real life situations that allow the development of robust gait recognition approaches, we propose a new framework, called Multiple Person Gait framework (MuPeG). This framework produces augmented datasets with multiple subjects in the scene by taking advantage of existing gait datasets. Thus, MuPeG combines an arbitrary number of subjects from existing datasets and creates new realistic video sequences that contain several subjects. By this way, videos with multiple subjects can be generated without recording a new dataset, what is a tedious and slow process. Moreover, our framework can produce different situations such as people walking beside or crossing each other, what produces multiple kinds of occlusions. Consequently, it can generate gait sequences more similar to real situations, setting up a new kind of benchmark that, to the best of our knowledge, did not exist before. Finally, to measure the suitability and difficulty of the generated dataset, we propose an experimental methodology composed of two types of experiments that we recommend to perform in datasets generated with our framework. The first one focuses on validating the video suitability of the generated dataset, and the second one focuses on measuring the performance of gait recognition approaches in scenes with multiple persons.

Thus, the main contributions of this paper are: *(i)* to propose and develop the first framework to produce augmented gait datasets with multiple subjects in the scene from existing datasets; *(ii)* to propose an experimental methodology for analyzing gait recognition datasets with multiple subjects in the scene; and, *(iii)* to perform the first accuracy evaluation of a gait recognition approach for all subjects appearing in the scene.

The rest of the paper is organized as follows. Section 2 summarizes the related work. In Section 3, we describe our framework together with the experimental methodology. Then, Section 4 contains the results of the proposed experiments, where the discussion of those results is carried out in Section 5. Finally, we present the conclusions in Section 6.

## 2. Related Work

In recent years, many approaches have been proposed for solving the problem of identifying people based on gait recognition [3] using different sources of data, such as inertial sensors [8,9], foot pressure [10], infrared images [11,12] or traditional images [13]. Most gait recognition studies use a stack of binary silhouettes as input data. In this regard, Gait Energy Image (GEI) [14] is the most popular silhouette-based gait descriptor. This descriptor is the result of computing a temporal averaging of the binary silhouette of the target subject. In contrast to descriptors based on silhouettes, Castro et al. [15] propose a method that uses dense local spatio-temporal features and a Fisher-based representation rearranged as tensors. Another example without silhouettes is the work carried out by Preis et al. [16], which uses a Kinect camera with an integrated depth sensor for skeleton detection and tracking in real-time.

The advent of Deep Learning (DL) architectures [17] has started a new realm of the feature learning field for recognition tasks. This trend is also observed in the gait recognition field and many DL research works have appeared in the last years. In Reference [18], Hossain et al. extract gait features from binary silhouettes using Restricted Boltzmann Machines. However, they use a small probe set (i.e., only ten subjects) for validating their approach. Yan et al. [19] extract high-level features that are used in a multi-task framework, where the goals are gait, angle view and scene recognition. They use as input data for a Convolutional Neural Network (CNN) the GEI descriptors computed on complete walking cycles. In Reference [20] the authors propose a CNN that accumulates the obtained features, in order to obtain a global representation of the dataset, using a random set of binary silhouettes of a sequence. In Reference [21], authors use raw 2D GEI to train an ensemble of CNNs using as classifier a Multilayer Perceptron (MLP). Similarly, in Reference [22] a multilayer CNN is trained with GEI data. In addition, in Reference [23], the authors developed a new approach based on GEI, where they train a CNN using pairs of gallery-probe samples. In Reference [24] the authors propose a different approach to the previous ones: the use of optical flow as input for training a CNN for gait recognition, obtaining state-of-the-art results. Despite most CNNs use visual data as input (e.g., images or videos), there are some works that build CNNs for different kinds of data like inertial sensors [9] or human pose [25]. Holden et al. [26] propose a CNN that corrects wrong human skeletons obtained by other methods or devices (e.g., Microsoft Kinect). Neverova et al. [27] build a temporal network for active biometric authentication with data provided by smartphone sensors (e.g., accelerometers, gyroscope, etc.).

However, all those approaches use datasets where the scene is completely controlled, that is, there are no occlusions and there is only one subject in the scene at the same time. It is important to note that static and dynamic occlusions will produce different problems. While the first is an obstacle, the second involves modifications in the calculation of predictors based on movement, such as optical flow, stack of silhouettes or GEI. Those datasets are summarized in Table 1. We briefly explain their main characteristics. *AVAMVG* [28] is characterized by the curved trajectories performed by the subjects and the occlusions of the lower part of the body. *CASIA-B* [29] is a cross-view gait dataset with people walking under different conditions (normal, carrying a bag or wearing coats). *CASIA-C* [30] is a dataset collected by an infrared camera and recorded during the night, with 5 different gait speeds per subject. The *CMU Mobo* [31] dataset has been recorded using six cameras with subjects walking on a treadmill and performing 4 types of step: slow, fast, inclined and walking with a ball. *OU-ISIR* [32] contains one of the largest number of subjects including a wide range of ages, from 1 to 94 years. *OU-ISIR MVPL* [33] is another dataset that contains a large number of subjects which recorded from multiple points of view (14 viewing angles). *Soton dataset* [34] is recorded with a time difference of 13 months between samples of the same subject, with the aim of isolating the effect of elapsed time in gait recognition. *TUM-GAID* [35] combines audio, RGB and depth signals to further study multi-modal gait recognition. *TUM-IITKGP* [7] dataset is traditionally used to develop gait recognition techniques to cope occlusions introduced in a subject. Finally, the *USF dataset* [23] is one of the most widely used gait databases and contains two fixed recording periods, collected outdoors with complex backgrounds.

Previous datasets do not contain any type of people occlusion, with the exception of TUM-IITKGP dataset [7]. It also contains multiple subjects in the scene, but they only try to identify the main subjects, considering others as occluding items. Therefore, gait recognition methods for multiple classifiable subjects are not studied. Using this specific dataset, several studies regarding how occlusions affect gait recognition have been performed. In Reference [4], they artificially degrade the visual quality of video sequences following three different frame degradation distributions. Then, perturbed silhouettes are reconstructed using the Balanced Gaussian Process Dynamic Model (BGPDM). Lately, the same authors [5] generate static and dynamic occlusions with different size, position and number of frames.In Reference [6], artificial (salt and pepper), static and dynamic occlusions are generated. With salt and pepper occlusions they simulate changes in the background of the scene. In the static occlusions, an object is inserted in the lower area of the image, which contains the most relevant information about the step. Finally, in dynamic occlusions, a moving object distorts the human silhouette.

In contrast, in our work, the occluding object is a person (i.e., it is dynamic and may eventually occlude the whole body of the other subject).

Other papers focus on reducing the impact of noise caused by failures in the segmentation of the silhouettes or by the clothing used by the subjects. Iwashita et al. [36] proposes to divide the GEI area into five horizontal areas to reduce the impact of the segmentation errors. References [37,38] focus on reducing the influence of clothing worn by subjects on the gait recognition accuracy. The first paper ([37]) identifies the direction of the gait samples and uses it to choose between gallery sets for doing the classification. For dealing with the occlusions of the clothes, it compares samples in that gallery with the GEI sample sections that are considered unaffected by clothing. The second paper ([38]), which uses an algorithm based on the golden number, separates for each subject the critical zone for gait recognition of the areas with clothes. To do this, the authors divide each training sample into six areas, each one with a different golden ratio, and they use them to train a gait recognition classifier.

Thus, one of the most common drawbacks in all these approaches and used datasets is that they only allow the study of scenes that contain a single subject at a given time. Therefore, when these datasets are used, factors such as the crossing between subjects or the prolonged overlap between them are not taken into account. As a result, most of the results obtained with those datasets cannot be extrapolated to real life situations with occlusions or multiple persons in the scene.

## 3. Methodology

Nowadays, current gait datasets contain only one identifiable subject in the scene at the same time. However, in a real life scenario, there can be multiple persons in the scene walking together, producing occlusions ones over others. Therefore, in order to measure the performance of gait recognition approaches under real life conditions, it is necessary to create a new dataset including multiple persons in the scene at the same time.

In this section, we propose a new framework for generating datasets with multiple subjects in the scene and an experimentation methodology that proposes how to evaluate the generated datasets.

### 3.1. MuPeG: Multiple Person Gait framework

We propose a framework, MuPeG, which is available in https://github.com/rubende/cnngait_tf, that combines subjects from a real dataset into the same scene. By this way, it is not necessary to record new sequences, as real subjects are synthetically included in the scene. Note that, since the proposed framework only needs videos originally published in these datasets, our framework can be potentially applied to any gait dataset published in the literature.

**Figure 1:**
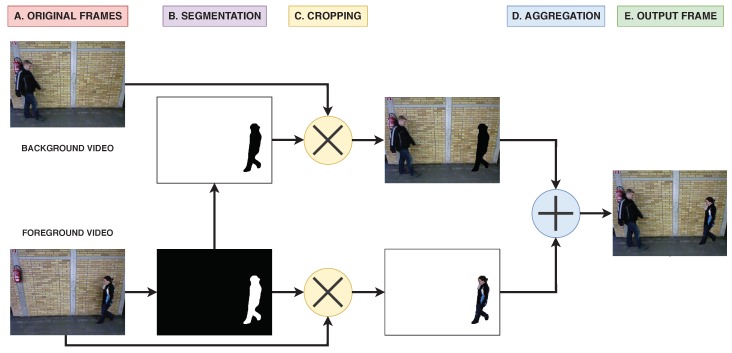
Multiple Person Gait framework (MuPeG) framework. (**A**) The inputs are two frames belonging to videos of different subjects. (**B**) Binary mask of the lower frame is obtained by segmentation. At the same time, the negative mask is computed for the upper frame. (**C**) Cropping operation is computed as the element-wise product of the masks by the corresponding frames. (**D**) Both frames are aggregated with an element-wise add operation. (**E**) The final output contains both subjects.

Our proposed framework consists of four main operations, as shown in Figure 1:

1. *Video selection.* In this step, we select the two videos that will be combined into a single scenario. The first video, the background video, is used as basis to add the second subject synthetically. Two considerations are taken into account in this step, the first one is to select videos from different subjects to avoid non realistic situations were the same subject appears twice in the scene. The second one is to use as background video those ones where the segmentation step described below tends to produce poor results due to the walking conditions (e.g., wearing coats, carrying objects, etc.). This selection can be seen in Figure 1A.
2. *Segmentation.* The goal of this step is to obtain the regions of the second video, foreground video, where the subject is located in each frame. Thus, the output of this step is a set of binary masks with the silhouette of the subject. This is a critical step since the suitability of the generated dataset depends on the suitability of the segmentation. Therefore, in order to obtain the best possible results, we use the state-of-the-art CNN segmentation model called *DeepLabV3+* [39], pretrained using *Pascal VOC 2012* dataset [40]. An example of the output obtained in this step is shown in Figure 1B. Note that the negative mask is obtained from the mask of the foreground video.
3. *Cropping.* Once the binary masks of both videos are generated, the background is cropped from the images in order to easily combine the foreground video with the background video used as basis. For that purpose, we just compute the element-wise product of the frames by the corresponding binary masks, producing new frames with black areas as shown in Figure 1C.
4. *Aggregation.* Finally, both videos are combined. This is an easy operation since a simple element-wise add operation is applied between the cropped frames of both videos as seen in Figure 1D,E.

**Figure 2:**
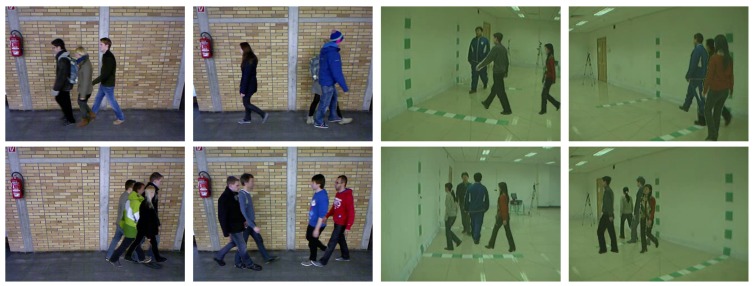
Samples generated with MuPeG. Output frames obtained with subjects from TUM-GAID and CASIA-B datasets under different walking conditions (view-points, carrying conditions, etc.).

Figure 2 shows some examples obtained with our framework. The first two columns are obtained from TUM-GAID [35] and the last two columns are obtained from CASIA-B [29]. As we can see in the images, MuPeG can be used in multiple scenarios with different view-points, walking conditions, and so forth. Moreover, it is possible to combine many subjects in the same scene.

### 3.2. Experimental Methodology

**Figure 3:**
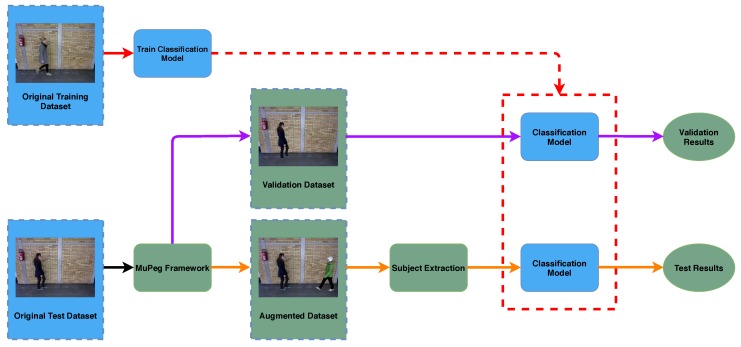
MuPeG use case. Outline of a typical use case of MuPeG framework. *Blue* elements identify training and test data belonging to the original dataset and the training of the Convolutional Neural Network (CNN) model based on the original training data. *Green* elements refer to procedures proposed in this paper. *Red* lines denotes the training process to obtain the Classification Model. *Purple* lines show the validation with just an aggregated subject. *Orange* lines represent the test with the augmented dataset including multiple subjects. (Best viewed in color).

We propose an experimental methodology for using gait datasets generated with our framework. This methodology is composed of two experiments that we recommend to carry out. The first experiment focuses on validating the suitability of the generated videos, and the second experiment is in charge of measuring the recognition capabilities of the models when multiple subjects are present in the scene. Note that this is an initial set of experiments and many different ones can be performed depending on the kind of dataset. Figure 3 shows an overview of our methodology. On the one hand, the original training data is used to generate a classification model. This process is represented by red lines. In fact, any model already trained for these datasets could be used. On the other hand, MuPeG is applied to the original test set so that both validation videos (purple lines) having just one aggregated subject and augmented videos (orange lines) containing multiples subjects are generated. Then, we use the classification model with the information generated with our framework to perform two different experiments as follows:

1. The goal of the first experiment, represented by purple arrows in Figure 3, is to validate the suitability and correctness of the augmented dataset. Thus, MuPeG is used to generate videos from the original test dataset with just one subject per video. These new videos constitute the validation test dataset, as indicated in Figure 3. By this way, the generated videos contain the same information than the original test dataset, but using segmented/aggregated subjects with MuPeG. Now, those videos are tested using a model trained on the original training set. The obtained test accuracy should be close to the accuracy obtained on the original test videos. If this condition is satisfied, we can assume that MuPeG is able to generate accurate augmented videos and more complex experiments, as the following one, could be performed.
2. The goal of this second experiment, path with orange arrows in Figure 3, is to measure the accuracy of gait recognition approaches under multiple subject conditions. For this experiment, gait recognition approaches are trained with the training data included in the original dataset, like the previous experiment, but now, they are tested on the augmented test dataset, also shown in Figure 3. It is necessary to apply a subject extraction, which allows us to maintain the relationship between the subjects and themselves throughout the sequence, with the aim of building the individual movement information. In order to extract valuable information from accuracy results, we recommend to split test samples into two categories: samples with subjects walking in the same direction and samples with subjects walking in opposite directions. By this way, it is possible to know the performance of gait recognition approaches under different levels of people overlap, i.e., when the subjects walk in the same direction occlusions stay longer, while when they walk in different directions, overlap uses to happen just during subject crossing and, consequently, takes shorter time.

## 4. Experiments

### 4.1. Datasets

We use our MuPeG framework to generate two augmented datasets using as basis two of the most widely used gait datasets—TUM-GAID [35] and CASIA-B [29]. Note that our framework needs datasets including RGB videos as the intended output of our framework is RGB videos.

Thus, the goal of those datasets is to measure the accuracy of gait recognition approaches in situations with multiple people, where the presence of multiple subjects simultaneously complicates the classification task. In this paper we only focus on two subjects in the scene to avoid excessive occlusion, since the recorded area is small and more subjects would overlap in most of the frames. In order to test all possible situations, we generate three kinds of videos: (a) two subjects walking together from left to right, (b) two subjects walking together from right to left and (c) two subjects walking in different directions. In all cases, both videos start at the same time, that is, from the first frame until the last one. By this way, we ensure that all subjects appear in the scene during enough time to be identified. Note that the length of each original video can be different because of the walking speed of each subject. However, in our combined videos, the length is the largest one and, if a subject walks faster than the other, it will disappear from the scene when its video is finished. Finally, in order to produce realistic videos, we do not allow the same subject to appear twice in the same combined video, since it is impossible in real life.

The main characteristic of the two augmented datasets can be summarized as:

1. *MultiTUM-GAID dataset*. This augmented dataset, based on TUM-GAID dataset, contains only test samples, since the training samples are the same ones used for training in TUM-GAID. The test video sequences of the new dataset, called augmented test dataset in Figure 3, are built using our framework MuPeG by merging two original test videos randomly chosen from TUM-GAID dataset. As we generate only test sequences, we focus on videos labeled as N5, N6, B1, B2, S1 and S2, which are the sequences belonging to the test set in TUM-GAID. In order to avoid segmentation problems caused by backpacks or coating shoes, only N5 and N6 videos are used as foreground. For the background, all videos are used. Overall, the augmented dataset used as test is composed of 155 subjects with a total of 1860 videos. Since the combination of subjects is performed randomly, the number of occurrences per subject differs between 16 and 34 videos, being the average 24 videos per subject.
2. *MultiCASIA-B dataset*. This second augmented dataset is based on CASIA-B dataset and, like with *MultiTUM-GAID*, we only generate test samples because the training samples are the same ones used for training in CASIA-B. The test video sequences of this new dataset, called augmented test dataset in Figure 3, are built using our framework MuPeG by merging two original test videos randomly chosen from CASIA-B dataset. Note that following the experimentation setup proposed in References [23,24], the first 74 subjects with all cameras are used for training and the last 50 subjects are used for testing. Thus, we focus on videos labeled as nm-5, nm-6, bg-1, bg-2, cl-1 and cl-2 for the last 50 subjects and the $90^{\circ }$ camera. Again, to avoid segmentation problems caused by backpacks or coats, only ‘nm’ videos are used as foreground. For the background, all videos are used. Overall, the augmented dataset used as test is composed of 50 subjects with a total of 600 videos. As in TUM-GAID, the combination of subjects is performed at random. The number of occurrences per subject differs between 17 and 36 videos, with the same average of 24 videos per subject.

**Figure 4:**
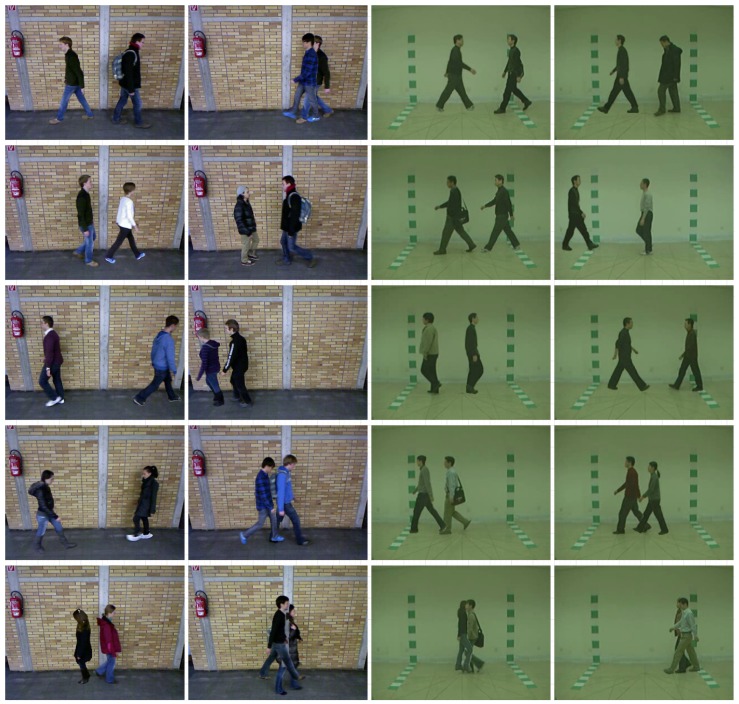
MultiTUM-GAID and MultiCASIA-B datasets. Different situations produced by our framework. Top four rows show frames generated with different subjects and different walking paths, exhibiting the quality of the output data. Bottom row shows some segmentation errors that produce lower quality outputs. The two leftmost columns show samples generated from TUM-GAID. The two rightmost columns are generated from CASIA-B.

Some samples of the augmented datasets can be seen in Figure 4. As we can see most of the frames are perfectly generated thanks to a robust segmentation process. Only in some cases shown in the bottom row problems appear, specially when some subjects walk much closer to the camera than the rest of the subjects (middle frame). In the other cases, the segmentation can produce some blank spaces of excessive borders that occlude the background subject.

### 4.2. Gait Recognition Approach

**Figure 5:**
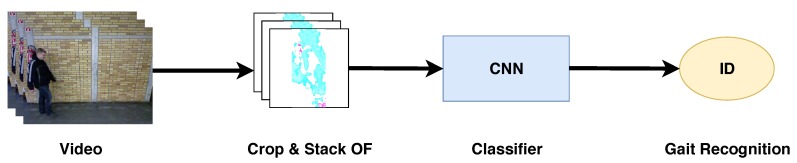
Pipeline for gait recognition. Pipeline based on References [24,41]. The input is a sequence of RGB frames where optical flow is calculated. Then, optical flow is cropped and stacked in 25-frame subsequences, keeping the subject centered in the 13th frame. Finally, the subsequences are passed through a CNN and classified by a SoftMax layer.

In this section we describe the gait recognition approach used in our experiments, which is based on References [24,41] but changing the people detection and tracking process since we have more than one subject in the scene. The approach presented in that paper reported state-of-the-art results on the original TUM-GAID and CASIA-B datasets. Thus, in this paper we only explain the main differences with regard to References [24,41], while the remaining details can be found in References [24,41]. Figure 5 summarizes the pipeline of this approach where, from the input video, we compute the optical flow maps. Then, 25 consecutive optical flow maps are stacked together and cropped to keep the subject in the middle of the 13th frame. Finally, the state-of-the-art CNN described in References [24,41] is trained. This CNN constitutes the classification model (see Figure 3) employed in experiments.

In order to find the position of the subjects in the scene, we use an object detector that includes the class ‘person’. In our case, we use the deep learning model called *Faster-RCNN-Inception-V2* [42], which is a well-known object detection CNN model. This model produces a set of bounding-boxes of the detected objects as seen in Figure 1B. Since we only need the position of the persons, we only keep those bounding-boxes belonging to that class. Note that in the original paper, the authors used a Gaussian Mixture model (GMM) to segment the background and obtain the position of subjects.

**Figure 6:**
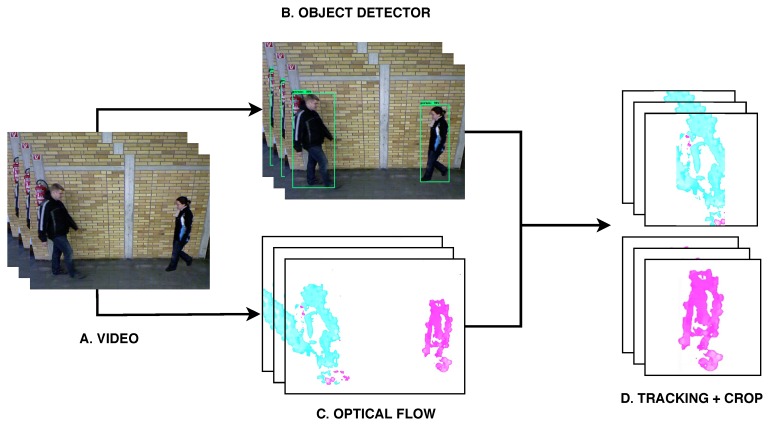
Input data generation. (**A**) The original data are sequences of video frames. (**B**) Persons appearing in the frames are detected and localized using an object detector. (**C**) Optical flow is computed for each pair of frames. (**D**) For each subject, optical flow maps are cropped and combined to produce a common output.

As explained above, once that we obtain the bounding-boxes of the subjects, we track the bounding-boxes of the same subject along 25 frames in order to crop the 80×60 optical flow maps to 60×60. By this way, we remove unnecessary information of the background and we limit the input size of the image as seen in Figure 6D.

Since the bounding-boxes produced by the object detector do not have any associated identity, we have to group them according to the subject they belong. In order to do this, we group similar bounding-boxes according to their similarity in a feature space obtained from a pretrained *ResNet50* model [43]. Thus, we feed the RGB information contained in each bounding-box to obtain a feature vector that describes that region. Finally, we group the bounding-boxes of each subject computing a L2 distance between the bounding-boxes of the current frame and the bounding-boxes of the previous frame. Then, the closest regions will be grouped together and at the end we obtain two groups of regions, one per subject.

Finally, using the bounding-boxes calculated above, we crop the optical flow maps according to their position. Notice that optical flow is calculated on augmented videos, so those obtained values can be very different from those of the original videos, specially when the subjects overlap each-other or if the process of building augmented videos does not work properly. Like in References [24,41], the crops are performed taking into account that in the central frame of the subsequence (frame 13), the horizontal centroid coordinate of the subject must be in the middle of the image.

### 4.3. Implementation Details

We performed our experiments on a server with two Xeon E5-2698 16 core processors, 256 GB of RAM and a NVidia Titan X. To develop our model, we used Keras [44] and Tensorflow [45] for Ubuntu 18.04.

The CNN architecture used in this paper is the same released in References [24,41], which is composed of four convolutional layers and two fully-connected layers. During training, we use standard Stochastic Gradient Descent (SGD) with mini-batches of 128 samples. The learning rate is set to 0.01 and divided by 10 when the validation loss does not improve in 3 epochs. Weight decay and momentum are set to 0.00005 and 0.9, respectively. We apply L2 regularization to the weights of the convolutional layers and dropout of 40% to the last two fully-connected (FC) layers.

Note that all the hyper-parameters have been cross-validated on the validation set before performing the final experiments.

### 4.4. Description of Experiments

As explained before in Section 3.2, two experiments have been carried out in the generated datasets, together with an additional experiment that focuses on measuring the impact of the overlap between subjects in the accuracy metric (see Equation (1), where *TP* is the number of true positives and *TN* is the number of true negatives):

(1) $$Acc=\frac{TP}{TP+TN}.$$

**Figure 7:**
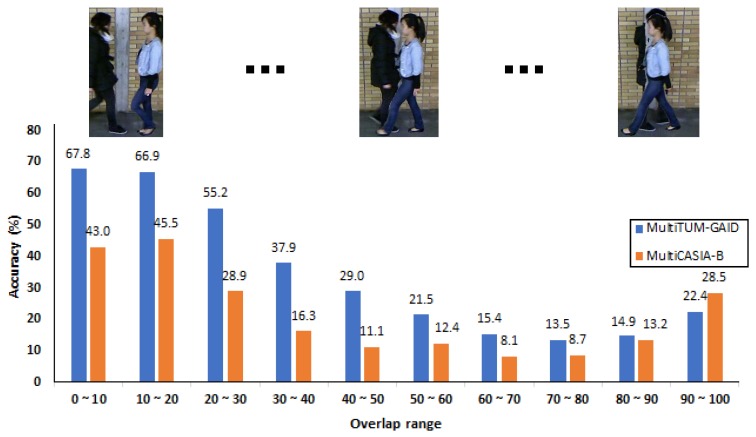
Accuracy under different grades of overlap. Each bar represents the accuracy of a different grade of overlap of the subjects present in a sample. More details in the main text. *Blue* bars correspond to TUM-GAID results, while *orange* bars correspond to CASIA-B results.

1. *Dataset validation.* For this purpose, we generated the whole test set of the original TUM-GAID and CASIA-B datasets using our framework but with only one subject in the scene. Thus, these validation datasets will contain the same subjects than the original ones but they have been synthetically added into the scene using a common background image found in the original datasets. Finally, those videos are fed into the CNN model proposed in References [24,41] which has been trained with the original training data included in each original dataset. Thus, if the augmented datasets are correctly generated, the accuracy achieved by the model should be similar to that obtained with the original test datasets.
2. *Multiple person recognition.* In this experiment, the gait recognition approach is trained with the training dataset included in each original dataset, like in the previous experiment, but now, it is tested on the augmented test datasets generated by MuPeG with multiple subjects in the scene. By this way, we are able to measure the generalization capabilities of the model to real life situations where multiple subjects are present in the same scene at the same time. In order to measure the performance of gait recognition approaches, we use the standard accuracy metric computed at video level, that is, combining the sample labels with a majority voting strategy. Thus, for each video, we will compare the two subjects predicted by the approach and the two ground-truth labels. Finally, in order to measure the accuracy per walking condition, we separate test samples according to their conditions like in the original TUM-GAID and CASIA-B datasets. The results obtained for this are summarized in Table 2 and Table 3, where each row represents a different approach. Two metrics are employed to measure the achieved performance. Thus, in the metric called ‘Subject level’, we consider each subject of the video individually in order to compute the accuracy. Similarly, with the metric called ‘Group level’, all subjects of the same video must be well-identified in order to consider the video as correctly classified. Regarding the columns of the table, ‘Walking direction’ contains the results obtained by splitting the test samples according to the walking patterns of the subjects: walking in different directions (‘Opposite’) and walking in the same direction (‘Same’). On the other hand, ‘Walking scenario’ shows the obtained accuracy when test samples are split according to the kind of walking conditions: normal gait (‘N’), carrying bags (‘B’) and wearing coating shoes (‘S’) for MultiTUM-GAID and normal gait (‘nm’), carrying bags (‘bg’) and wearing coats (‘cl’) for MultiCASIA-B. Finally, ‘Global’ column indicates the accuracy for all test samples. The discussion of those results is carried out in the following section.
3. *Person overlap*. Finally, in this additional experiment, we measure the accuracy of the model used in the previous experiment but, in this case, according to the overlap of the subjects in a sample. In order to measure the overlap of the samples, we compute the average intersection area (intersected area divided by the area of the smallest bounding-box) of both bounding-boxes along the 25 frames of a sample. Then, once the test samples are characterized according to the intersection, we obtain the accuracy of the model per sample of 25 frames. Figure 7 contains the results for this experiment, where the horizontal axis represents the overlap range and the vertical axis represents the accuracy at sample level. Note that, like in previous experiments, the results are obtained at sample (stack of 25 frames) level instead of video one since at video level the intersection between people is lower and the results would be condensed in a few bins. The discussion of these results is carried out in the following section.

## 5. Discussion

In this section, we comment on the results obtained for the experiments described in Section 4.4.

### 5.1. Dataset Validation Experiment

We discuss here the first experiment, that is, the validation of the dataset using videos generated with a single subject in the scene. Following the proposed indications in Section 4.4, we use a CNN trained with the training set of original videos included in TUM-GAID and the performance evaluation is carried out with the validation dataset generated by MuPeG. In this experiment, we achieve an accuracy of 98.0%. When the same model is applied to test data of the original dataset, the obtained accuracy is 99.7%. Similarly, we perform the same training and validation process with CASIA-B dataset. In this case, using the augmented videos we obtain an accuracy of 97.0% while using the original videos we achieve a 98.0%. Therefore, we can assume that our framework produces datasets that look realistic, since a model trained with the original dataset and tested with our augmented dataset with one subject obtains an accuracy score very close to the one obtained on the original test dataset.

### 5.2. Multiple Person Recognition

Once the framework is validated, we move to the second experiment using multiple persons in the scene. The results of these experiments are summarized in Table 2 and Table 3. Focusing on the first approach (‘L2Tracker+CNN’), see Section 4.2, and comparing the results obtained for the normal scenario (‘N’ in MultiTUM-GAID, ‘nm’ in MultiCASIA-B) at subject level in experiment one (one subject in the scene) and experiment two (two subjects in the scene), we can see a big drop in the performance, from 98.0% to 57.8% in MultiTUM-GAID and from 98.0% to 59.1% in MultiCASIA-B. In our opinion, this decrease in the performance is the consequence of two main facts: the overlap among subjects, which produces wrong optical flow vectors, and the performance of the tracker, that confuses the bounding boxes of the subjects and constructs optical flow windows with errors, mixing the subjects in them. Focusing on the different metrics, we can see that the accuracy is higher at subject level than at group level. However, the behaviour among scenarios is similar in both cases and the difference is due to the stricter metric for the group level, as all subjects in a video must be classified correctly. If we focus on the ‘Walking direction’ column, we can observe that the classification is clearly worse when the subjects walk in the same direction. This is because in this situation both subjects overlap each other, sometimes during few frames and other times during the whole video, what makes more difficult to classify these subjects. In MultiCASIA-B, we have observed that this problem is less frequent since the subjects walk with many different speeds so they do not overlap as much as in MultiTUM-GAID, where the subjects have similar walking speeds. Concentrating on the ‘Walking scenario’, we can observe that the results of the normal scenario (‘N’ from MultiTUM-GAID and ‘nm’ for MultiCASIA-B) are better than the other kind of walking conditions, which coincides with the behaviour already observed in References [24,41]. Thus, it can be explained taking into account that during the training of the classifier, there are only normal samples (‘N’ or ‘nm’ depending on the dataset) and the model tends to forget how to deal with the other different scenarios. Comparing the ‘Global’ accuracies between MultiTUM-GAID and MultiCASIA-B we can see a clear drop in MultiCASIA-B, mainly due to the ‘cl’ scenario that is very challenging since it introduces long coats.

In order to corroborate our intuition regarding the performance of the tracker, we have designed an additional experiment considering a ground-truth tracker that never fails. Since tracking is performed during the segmentation process of our framework (see Section 3.1 for more details), we can build a perfect tracking just grouping together, during subject aggregation, bounding boxes belonging to the same subject, avoiding, in this way, the confusion between them. Note that this is not a fair tracker because it is designed only for validating our hypothesis and cannot be used in real scenarios. Comparing the results of this approach (‘GroundTruthTracker+CNN’), for both datasets, with the previous ones, we can see a clear improvement in both metrics, specially for the ‘Opposite’ case. Therefore, a better tracker improves the identification performance of our approach. However, for the ‘Same’ case, the improvement is very limited because of the subject overlapping problem (shown in Figure 4), which cannot be solved by this ground-truth tracker. Moreover, since our CNN uses 60×60 pixels input sample, if subjects walk close in the scene without overlapping, the input sample could contain data coming from both subjects, confusing the classifier. In order to deal with this case, we propose a cropping process for each detected subject that removes other subjects appearing in the scene. This process uses the bounding-box of the subject as a Region of Interest (ROI) and removes the optical flow vectors located outside of this ROI. By this way, we remove other subjects appearing in the scene. Comparing the results for this new version (‘Cropping+L2Tracker+CNN’ and ‘Cropping+GroundTruthTracker+CNN’) with the previous ones, we can see that only for the ground-truth tracker there is an improvement of the results. In our opinion, the reason is that if the tracker fails, the cropping process might remove critical information, decreasing the accuracy. Thus, since the ground-truth tracker never fails, the results can be improved, specially in the ‘Same’ case. In real situation, if the used tracker does not provide reliable results, it is better to avoid the cropping strategy.

### 5.3. Person Overlap

Finally, focusing on the last experiment, we measure the accuracy of the ‘GroundTruthTracker+CNN’ under different grades of overlap among subjects for both datasets. Note that we use this version of the tracker with the aim of providing correct bounding boxes of the same subject to the model. This way, our experiment focuses on errors produced just by occlusions. The results of this experiment are summarized in Figure 7. We use the accuracy at sample level, using 25 frames length windows, since we are measuring the overlap per sample. Focusing on the results, we can see that, as expected, the accuracy is high (67.8% in MultiTUM-GAID and 43.0% in MultiCASIA-B) with small overlap ranges and starts to decrease with larger overlaps. Thus, as subjects move closer, the overlapping area produces noisy optical flow values that reduce the accuracy score. However, it can be seen in the rightmost part of the plot that the accuracy improves, around 9% for MultiTUM-GAID and around 15% for MultiCASIA-B, from the range [70,80) to the range [90,100]. In our opinion, this can be explained taking into account that in situations with large overlaps, one subject is very visible in the scene, while the other one is almost hidden behind the foreground subject. Thus, the optical flow noise added by the background subject on the foreground one is small and does not penalize excessively the classification scores for the foreground subject. This situation happens more often when subjects walk in the same direction. Note that in MultiCASIA-B, the results are more unstable among overlap steps due to the extremely noisy background used in this dataset, specially due to the marks included in the wall, as seen in Figure 4.

## 6. Conclusions

In this paper, we have proposed the first framework (MuPeG) to generate augmented gait datasets with multiple persons in the scene using existing datasets. This framework allows researchers to build a new type of datasets that did not exist before in the state-of-the-art, opening new challenges for researchers, as all previous state-of-the-art datasets have only one subject per sequence and, consequently, they do not allow to deal with realistic gait analysis problems.

In order to use the datasets generated with our MuPeG framework, we have proposed an experimental methodology that defines the minimum number and type of experiments that must be performed in this kind of datasets. Specifically, we define two experiments, one for validating the suitability of the generated datasets and a second one, that defines how to perform the experiments under multiple person conditions.

Lastly, we use the approach proposed in References [24,41], which obtains state-of-the-art results in TUM-GAID and CASIA-B datasets, to obtain the first results for gait recognition with multiple persons in the scene. Comparing the results obtained in multiple subject datasets with the ones obtained in traditional ones, the difficulty of this kind of datasets is demonstrated, since the results drops around a 50%. In contrast, in single subject datasets, it is very common to see reported accuracy values higher than 90%. Two conclusions can be extracted from the results. On the one hand, the development of a good tracking algorithm to extract isolated subjects is very important so that the extracted samples can be classified correctly. Note that, the tracking process is necessary for all gait recognition methods since multiple frames are required to describe the gait movement. On the other hand, attending to experimental results it is necessary to train classifiers to deal with subject overlapping, specially when subjects walk in the same direction. Thus, the experiments have shown that the greater the overlap, the worse the results.

Finally, as future work, we plan to develop both new tracking approaches to improve the results proposed in this paper and better CNN models able to deal with multiple persons. Moreover, we will try to define more experiments to take advantage of the multiple person gait datasets generated with our framework.

## Figures and Tables

**Table 1:** Comparison of state-of-the-art gait datasets.

Name	Subjects	Views	Occlusions	Multi-Subjects	Time Span	Main Application
AVAMVG [28]	20	6	Yes	No	-	Cross-view and curved paths
CASIA-B [29]	124	11	No	No	-	Cross-view gait recognition
CASIA-C [30]	153	1	No	No	-	Night gait recognition
CMU Mobo [31]	25	6	No	No	-	Gait analysis
OU-ISIR [32]	4016	4	No	No	-	Gait analysis
OU-ISIR MVPL [33]	10,307	14	No	No	-	Cross-view
Soton [34]	25	12	No	No	13 months	Gait recognition
TUM-GAID [35]	305	1	No	No	3 months	Multimodal gait recognition
TUM-IITKGP [7]	35	1	Yes	No	-	Gait recognition with occlusions
USF [23]	20	2	No	No	-	Gait recognition

**Table 2:** Accuracy on MultiTUM-GAID. Each row represents a different granularity of the experiment. Each column represents a different test scenario. Best global results are marked in bold. More details in the text.

Experiment	Metric	Walking Direction		Walking Scenario			Global
		Opposite	Same	N	B	S	
L2Tracker+CNN	Subject level	79.1	20.3	57.8	52.8	53.5	54.7
	Group level	61.3	3.0	36.3	30.2	30.5	32.3
GroundTruthTracker+CNN	Subject level	94.2	27.5	61.6	58.6	62.4	60.8
	Group level	89.3	3.7	47.7	44.2	47.6	46.5
Cropping+L2Tracker+CNN	Subject level	69.3	31.2	57.5	47.2	55.7	53.5
	Group level	45.1	14.0	34.5	23.4	31.2	29.7
Cropping+GroundTruthTracker+CNN	Subject level	95.2	49.0	74.8	68.1	73.5	**72.1**
	Group level	90.6	25.4	61.3	53.6	59.2	**58.0**

**Table 3:** Accuracy on MultiCASIA-B. Each row represents a different granularity of the experiment. Each column represents a different test scenario. Best global results are marked in bold. More details in the text.

Experiment	Metric	Walking Direction		Walking Scenario			Global
		Opposite	Same	nm	bg	cl	
L2Tracker+CNN	Subject level	51.3	40.3	59.1	53.4	40.8	45.7
	Group level	26.1	17.3	36.0	29.3	13.0	21.8
GroundTruthTracker+CNN	Subject level	75.3	47.2	91.0	80.5	54.5	61.3
	Group level	53.0	27.0	83.0	62.0	14.0	40.0
Cropping+L2Tracker+CNN	Subject level	45.1	37.1	50.9	48.4	35.5	41.0
	Group level	19.4	11.9	25.0	22.2	11.0	15.7
Cropping+GroundTruthTracker+CNN	Subject level	74.0	54.0	89.5	77.0	55.5	**64.0**
	Group level	51.0	33.7	79.0	57.0	17.0	**42.3**
